# The murine ATP-binding cassette transporter C5 (Abcc5/MRP5/cMOAT) plays a role in memory consolidation, circadian rhythm regulation and glutamatergic signalling

**DOI:** 10.1038/s41398-025-03438-9

**Published:** 2025-07-01

**Authors:** Gareth Banks, Malgorzata Cyranka, Natascia Vedovato, Charlotte Meller, Alex Rawnsley, Edward O. Mann, Michelle Stewart, Heidi de Wet

**Affiliations:** 1 School of Science and Technology, Nottingham Trent University, Nottingham, UK; 2 Dept of Physiology, Anatomy and Genetics, University of Oxford, Oxford, Oxfordshire UK; 3 MRC Harwell Institute, Mary Lyon Centre, Harwell Campus, Didcot, Oxfordshire UK; 4 Electron Bio-Imaging Centre (eBIC), Diamond Light Source and Research Complex at Harwell, Harwell Science & Innovation Campus, Didcot, Oxfordshire UK

**Keywords:** Neuroscience, Learning and memory

## Abstract

ATP-Binding cassette (ABC) transporters are a family of integral membrane ATPases that transport a large number of structurally unrelated compounds. The physiological role of the orphan transporter Abcc5 remains poorly understood. As previous work demonstrated that the loss of Abcc5 activity leads to elevated levels of NAAG in the brain, the impact of Abcc5 ablation was ascertained using behavioural phenotyping, circadian rhythm analysis and electrophysiological recordings of brain slices from *Abcc5*^*−/−*^ mice and compared to wild-type littermates. Behavioural phenotyping of *Abcc5*^*−/−*^ mice shows that the loss of murine Abcc5 activity results in profound changes in pre-pulse inhibition (PPI) as well as altered memory consolidation. Circadian measures of activity showed a delay in the timing of *Abcc5*^*−/−*^ mice activity rhythm peak. Additionally, activity defined sleep analysis highlighted differences in sleep patterns in *Abcc5*^*−/−*^ mice compared to wild-type controls. Patch clamp recording from pyramidal cells in the 2/3 layer of the frontal cortex showed altered synaptic AMPA/NMDA receptor current ratios and increased frequency of spontaneous excitatory postsynaptic currents (sEPSC). This study demonstrates that the loss of functional Abcc5 transporters does have behavioural consequences in mammals and alters NMDA receptor activity. These results highlight a previously unknown role of Abcc5 in the brain.

## Introduction

ATP-Binding cassette (ABC) transporters are a superfamily of integral membrane ATPases that transport many structurally unrelated compounds into and out of cells [1]. In mammals, ABC transporters are exporters and are most well known for their role in chemotherapy-resistant tumours [2].

The physiological role of Abcc5 remains poorly understood, but the overexpression of human ABCC5 is strongly correlated with multi-drug resistance [3]. Recombinant expression of murine Abcc5 and human ABCC5 protein in inside-out vesicles has shown that Abcc5 can transport cyclic nucleotide analogues, antifolates, folic acid and *N*-lactoyl-amino acids [4–6], but the primary physiological substrates of mammalian Abcc5 are most likely C-terminal glutamate dipeptides such as *N*-acetylaspartylglutamate (NAAG), *N*-acetylaspartyl-diglutamate (NAAG_2_), beta-citrylgluamate (BCG) and beta-citryldiglutamate (BCG_2_) [4]. NAAG, the third most common neurotransmitter in the mammalian brain, is an inhibitory neurotransmitter, and is generally accepted to act as an antagonist of excitatory glutamatergic signalling [7, 8]. Synaptically-released glutamate excites post-synaptic neurons through G-protein coupled receptors (mGluR) and ion channels (iGluR). NAAG exerts its effect through the activation of an inhibitory GPCR (mGluR3) and possibly through the regulation of a glutamate-activated ion channel, the *N*-methyl-*D*-aspartate (NMDA) receptor. Glutamatergic signalling could therefore be potentially impacted by the loss of Abcc5.

In humans, *ABCC5* gene overexpression is linked to a susceptibility to type 2 diabetes and increased visceral adiposity [9, 10]. Work from our laboratory showed that *Abcc5*^*−/−*^ mice had a clear metabolic phenotype, weighed less and were more insulin sensitive [11]. Furthermore, *Abcc5*^*−/−*^ mice were also more active in both the light and dark phase [11] suggesting a possible role of Abcc5 in behaviour and neurotransmission. A number of studies have also implicated Abcc5 in nervous function and behaviour, white matter structures in the brain, mental health traits, cognitive function, memory and depression [12–15]. While such studies are suggestive of a role for Abcc5 in nervous function, direct evidence of this is currently lacking.

Here we perform extensive behavioural phenotyping of a global *Abcc5*^*−/−*^ mouse strain (C57BL/6NTac-Abcc5^em1(IMPC)H^/H) to ascertain if the loss of Abcc5 expression does have neurological implications. Gene expression databases suggest that in the mouse brain, Abcc5 is expressed in a range of different brain regions and cell types [16]. Since this expression pattern did not highlight an obvious brain region or neuronal output which would be impacted by the loss of *Abcc5*, we investigated a broad range of behavioural phenotypes in the *Abcc5*^*−/−*^ mouse strain to identify which systems or behaviours would be affected by the loss of the gene. Our findings demonstrate that the loss of Abcc5 impacts on aspects of sensory motor gating, learning and memory, sleep and glutamatergic synaptic transmission. The data directly highlight a hitherto unknown function of Abcc5 within the brain.

## Methods

### CRISPR design of *Abcc5*^*−/−*^ mice

*Abcc5*^*−/−*^ CRISPR/Cas9 mice (C57BL/6NTac-Abcc5^em1(IMPC)H/H^) were generated on a C57BL/6NTac background in the Medical Research Council (MRC) Harwell Institute; European Mouse Mutant Archive (www.infra front ier.eu). CRISPR design and the topological overview of Abcc5 protein structure is shown in Supplementary Fig. 1A, B. The CRISPR protospacer sequences were *Abcc5_*5′, 5′-GCTGTGGGTTGCTGATTGCAGGG3′; and *Abcc5_*3′, 5′-CTTCTCTCACACATAGC CAAAGG-3′.

### Animal studies

Animal studies were performed under guidance from the Medical Research Council in Responsibility in the Use of Animals for Medical Research (July 1993), the University of Oxford’s Policy on the Use of Animals in Scientific Research, and Home Office Project License PP6741514 for the University of Oxford and 30/3384 for MRC Harwell. When not tested, mice were housed in individually ventilated cages under 12/12 h light/dark conditions with food and water available ad libitum. Researchers were blinded to mouse genotype in behavioural studies.

### Acoustic startle and prepulse inhibition (PPI)

Acoustic startle response and prepulse inhibition were measured as described in [17]. Briefly, mice were placed in a recording chamber (Med Associates, VT, USA), and responses to sound stimuli were measured via motion sensitive platforms. Animals were tested at 15 weeks of age.

### Fear conditioning

Fear conditioning was performed as described in [18]. Briefly, mice were placed in a fear conditioning system arena (Ugo Basile, Italy) and monitored for freezing behaviour using ANYMaze video tracking system (Stoelting Europe). Animals were tested at 16 weeks of age.

### Open field

Mice were placed into one corner of a walled arena (45 cm by 45 cm) and allowed to freely explore for 20 min [17]. Animal movements and position were tracked using EthoVision XT analysis software (Noldus). Animals were tested at 10 weeks of age.

### Grip strength

Grip strength was assessed using a Grip Strength Meter (BioSeb, Chaville, France) as described previously [18]. Readings were taken in triplicate from both forelimb only and combined forelimb and hindlimbs, before being averaged and normalized to body weight. Animals were tested at 14 weeks of age.

### Circadian and sleep monitoring

Mice were analysed for circadian activity and immobility-defined sleep using the COMPASS system as described [19]. Briefly, mouse movement was captured by a passive infrared sensor, with periods of immobility of 40 s or more scored as sleep. Data captured for 5 days in a (12 h:12 h) light/dark cycle, followed by 9 days in constant darkness. Analysis was performed using custom python scripts and Clocklab (Actimetrics). Animals were tested at 17 weeks of age.

### Electrophysiology and pharmacological manipulations

Mice were anesthetized using 4% isoflurane, decapitated and the brains extracted in ice cold NMDG solution (93 mM NMDG, 2.5 mM KCl, 1.25 mM NaH_2_PO_4_, 30 mM NaHCO_3_, 20 mM HEPES, 10 mM MgSO_4_.7H_2_0, 0.5 mM CaCl_2_.2H_2_0 and 25 mM glucose, with pH adjusted to 7.2–7.3 using 1 M HCl solution; osmolality 300 ± 10 mOsmol/kg). Coronal slices 350 μm thick were cut using a vibratome (Leica VT1200S) and transferred to a submerged chamber containing warm NMDG solution (32–34 °C). Slices were subsequently transferred to an interface chamber containing artificial cerebrospinal fluid (aCSF) (126 mM NaCl, 3.5 mM KCl, 2 mM MgSO_4_.7H_2_O, 1.25 mM NaH_2_PO_4_, 24 mM NaHCO_3_, 2 mM CaCl_2_, and 10 mM glucose; osmolality 300 ± 10 mOsmol/kg) at room temperature. For recordings, slices were transferred to a chamber continuously perfused with aCSF at 32–34 °C. All solutions were bubbled with carbogen gas (95% O_2_/ 5% CO_2_).

Whole-cell voltage-clamp recordings from pyramidal cells in the 2/3 layer of the frontal cortex were performed using glass pipettes with a resistance of 5–8MΩ when filled with 110 mM caesium methanesulfonate, 4 mM NaCl, 40 mM HEPES, 2 mM MgATP, 0.3 mM Na_3_GTP and 4 mg/mL biocytin (Sigma-Aldrich), pH adjusted to 7.3 with CsOH. Traces were sampled at 10 kHz using a Multiclamp 700B amplifier (Molecular Devices), InstruTECH ITC-18 analog/digital board (HEKA) and IGOR Pro software.

Pyramidal cells were voltage-clamped at −70 mV to measure excitatory post-synaptic currents (EPSCs). The activation of the cortical neurons was assessed using a bipolar electrode (100–200 μA, 100 μs), placed near the recording layer. Evoked EPSCs were recorded in the presence of blockers of inhibitory GABAergic transmission (10 μM bicuculline, Tocris Bioscience). Cells were then voltage-clamped at +40 mV and EPSCs recorded again. AMPA/NMDA receptor current ratios were calculated by dividing the average peak amplitude of evoked EPSCs at −70 mV (AMPAR current) by the average amplitude of evoked EPSCs at +40 mV, 5 ms after the stimulation (NMDAR currents). All buffers were sourced from Sigma-Aldrich.

For the detection of spontaneous EPSCs, the signal was low-pass filtered at 1 kHz, event initially detected as those with 2 consecutive points exceeding 7 pA, and events less than 5 SD above the noise rejected. To avoid multiple detections of large events, the event detection algorithm recommenced after the peak of each detected event.

### Statistics

All statistics were performed in GraphPad Prism and data are presented as mean ± SEM. For experiments with one independent variable, a Welch and Brown-Forsythe one-way ANOVA was used (as this test do not assume equal variances for wild-type (wt) and *Abcc5*^*−/−*^ (ko) mice) in combination with a Dunnet multiple comparison post hoc test. For experiments with two independent variables, where an unequal number of wt and *Abcc5*^*−/−*^ mice were used (for example, in the behavioural studies, females: n_ko_ = 15, n_wt_ = 17; males: n_ko_ = 15, n_wt_ = 13) a mixed-effect analysis combined with restricted maximum likelihood (REML) calculations was used in combination with Šidák’s multiple comparison post hoc test. For genotype comparison within the same treatment group, multiple comparisons were done using a Fisher’s least significant difference (LSD) test.

## Results

### *Abcc5*^*−/−*^ mice show an altered acoustic startle reflex (ASR) and decreased prepulse inhibition (PPI)

**Fig. 1:**
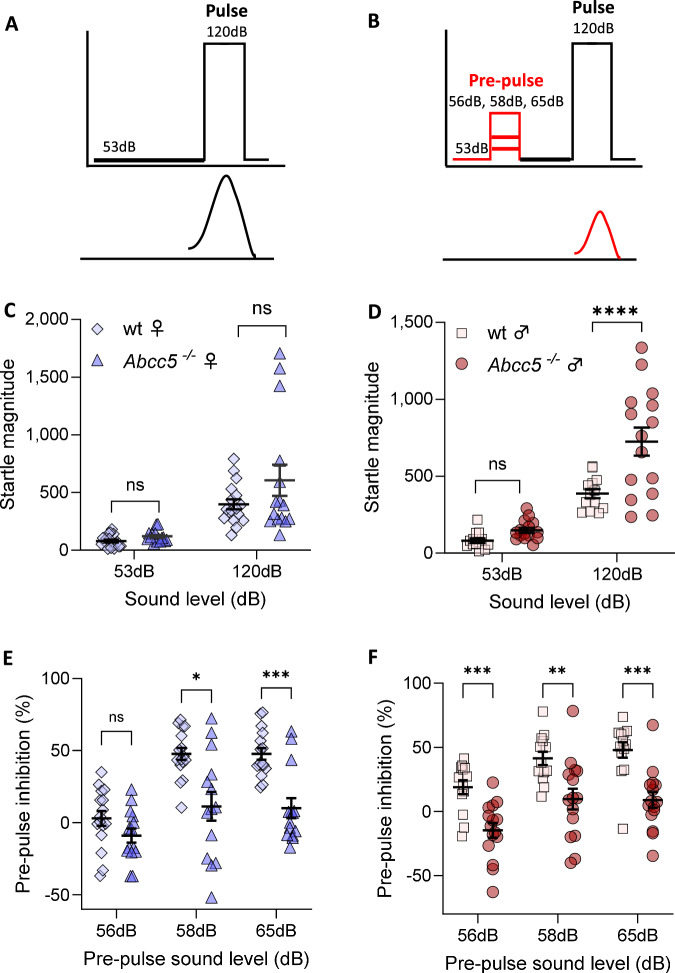
*Abcc5*^*−/−*^ mice show an altered acoustic startle reflex (ASR) and decreased pre-pulse inhibition (PPI). **A**, **B** Graphical description of the pre-pulse inhibition principle. **A** Startle in response to a single 120 dB pulse. **B** Applying a pre-pulse stimulus (red line) paired with the startle pulse (black line) will cause a decrease (inhibition) of the startle response. **C**, **D** The magnitude of the startle response to a background pulse (53 dB) and maximal startle pulse (120 dB) alone in wild type (wt) mice and *Abcc5*^*−/−*^ (ko) mice for **C** females (diamonds and triangles) and **D** males (squares and circles). Percentage of pre-pulse inhibition tested for 3 pre-pulse levels 56, 58, 65 dB measured for wt and *Abcc5*^*−/−*^ mice in **E** females and **F** males showing a dramatic loss of pre-pulse inhibition in *Abcc5*^*−/−*^ mice. Females: n_ko_ = 15, n_wt_ = 17 and males: n_ko_ = 15, n_wt_ = 13. Data presented as mean ± SEM and each data point represent an average of 10 measurements from a single mouse. Results were compared by mixed-effect analysis combined with a Fisher’s LSD test for genotype comparison in the same treatment group, and a Šidák’s multiple comparison test when comparing genotypes across treatments; fixed effects: dB, genotype and dB x genotype; random effects: individual mice and residuals; *P* < 0.05 *, *P* < 0.01 **, *P* < 0.001 ***, *P* < 0.0001****.

Prepulse inhibition (PPI) of the startle response is a reliable measure of processing deficits and inhibitory failure in schizophrenia patients as well as mouse models [20]. As glutamatergic signalling is potentially impacted by the loss of Abcc5, PPI was investigated in *Abcc5*^*−/−*^ mice and wild-type (wt) littermate controls. Female and male *Abcc5*^*−/−*^ mice and wild-type (wt) littermate controls were exposed to a baseline (53 dB) and a startle (120 dB) pulse to determine the baseline acoustic startle reflex (ASR) (Fig. 1A, C and D). The background tone (53 dB) elicited a similar response in both *Abcc5*^*−/−*^ females (Fig. 1C) and males (Fig. 1D) relative to wt controls. *Abcc5*^*−/−*^ males demonstrated higher reactivity to the 120 dB startle pulse relative to wt controls (726 ± 91 vs 386 ± 29.7, *P* = 0.0026, Fig. 1D), while *Abcc5*^*−/−*^ females showed a similar trend in startle reactivity, which was not statistically significant (606.3 ± 135 vs 397 ± 42, *P* = 0.131, Fig. 1C).

Next, PPI was measured in response to prepulses at different levels prior to the startle pulse of 120 dB (Fig. 1B, E and F). *Abcc5*^*−/−*^ mice and wt littermate controls were subjected to three prepulse trials of 56 dB, 58 dB, or 65 dB preceding the startle pulse of 120 dB. The startle reflex inhibition elicited by each prepulse level was calculated and plotted as the percentage of startle response alone. PPI in *Abcc5*^*−/−*^ females was significantly different when compared to wt for interaction (prepulse x genotype, *P* = 0.0025), prepulse (*P* < 0.0001) and genotype (*P* = 0.004) relative to wt controls (Fig. 1E). *Abcc5*^*−/−*^ females demonstrated significant decreases in PPI relative to wt controls at 58 dB (11.3% ± 9.9 vs 47.6% ± 4.2), and 65 dB (10.1% ± 6.9 vs 47.7% ± 4.0) respectively (Fig. 1E, *P* = 0.0106 and *P* = 0.004). PPI in *Abcc5*^*−/−*^ males were not significantly different when compared to wt for interaction (prepulse x genotype, *P* = 0.72), but were significant for prepulse (*P* < 0.0001) and genotype (*P* < 0.0001). *Abcc5*^*−/−*^ males demonstrated significant decreases in PPI relative to wt controls at all prepulse levels, 56 db (14.7% ± 5.8 vs 18.9% ± 5.4), 58 dB (9.6% ± 8 vs 41.4% ± 5.2) and 65 dB (8.9% ± 6.1 vs 47.9% ± 6.1), Fig. 1F (*P* = 0.0007, *P* = 0.009 and *P* = 0.0004).

It is important to note that all tested prepulse sound levels caused prepulse facilitation (PPF) effects in some *Abcc5*^*−/−*^ mice of both sexes which results in a negative percentage value of PPI (Fig. 1E, F). PPF occurs when a weaker prepulse is capable of enhancing the magnitude of the ASR response (instead of inhibiting it) and/or reducing the latency of the startle response [21, 22]. Interestingly, some wt controls of both sexes exhibited PPF at 56 dB, which was absent at 58 and 65 dB for all wt mice (Fig. 1E, F).

**Fig. 2:**
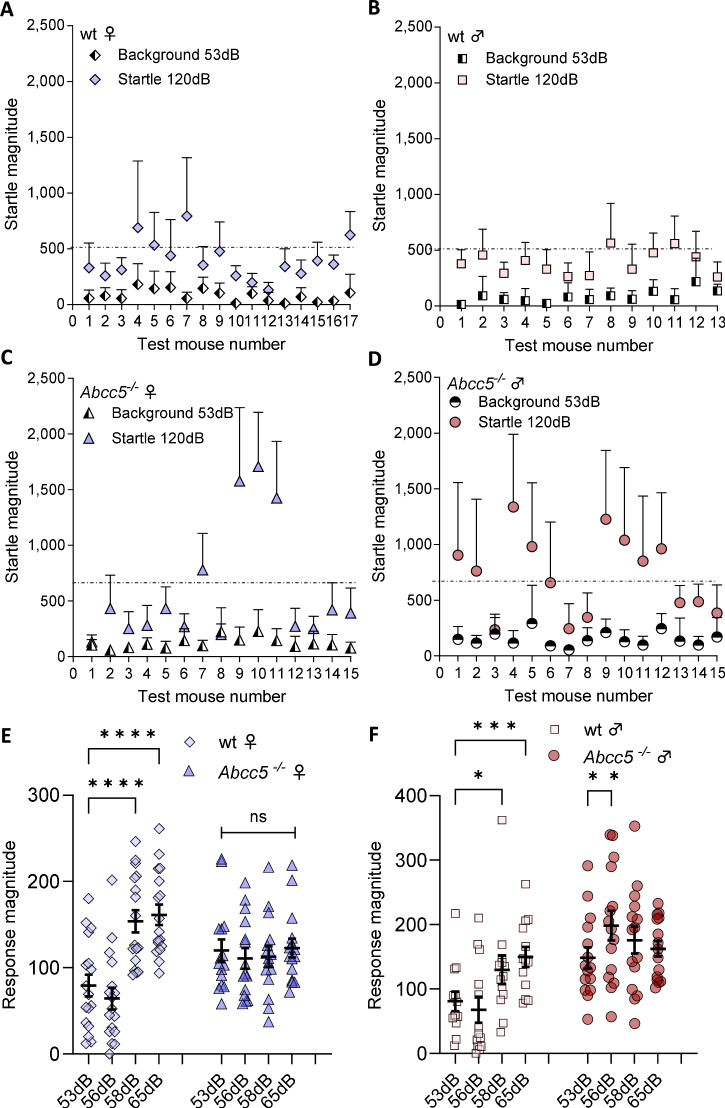
Comparison of background and startle responses obtained from individual mice. (**A**–**D** The startle magnitude of **A** wt females (diamonds), **B** wt males (squares), **C** *Abcc5*^*−/−*^ females (triangles) and **D** *Abcc5*^*−/−*^ males (circles). Data presented as a single point per mouse, error bars ± SEM. **E**, **F** The magnitude of the response to background noise (53 dB) and three different prepulse levels alone (56, 58, 65 dB) in *Abcc5*^*−/−*^ and wt mice in **E** females and **F** males. Data presented for **E** and **F** as mean ± SEM. Females: n_ko_ = 15, n_wt_ = 17 and males: n_ko_ = 15, n_wt_ = 13. Each data point represents an average of 10 measurements from a single mouse. Results for E and F were compared by mixed-effect analysis combined with a Šidák’s multiple comparison test; fixed effects: dB, genotype and dB x genotype; random effects: individual mice and residuals; *P* < 0.05 *, *P* < 0.01 **, *P* < 0.001 ***, *P* < 0.0001 ****.

In order to investigate the ASR and PPI reflexes in more detail, baseline responses to the background noise and the magnitude of startle reactivity were plotted for individual mice (Fig. 2A–D). The startle response in *Abcc5*^*−/−*^ mice was not as uniform as in wt littermates, as can be seen in the significantly increased SD in the *Abcc5*^*−/−*^ mice (unpaired t test, F test comparing variances, *P* = 0.001). Furthermore, 3 of the 15 *Abcc5*^*−/−*^ female mice tested had much higher startle magnitudes (mice 9, 10 and 11) and were confirmed to be statistical outliers (Fig. 2C). Variation in startle magnitude was markedly different in *Abcc5*^*−/−*^ male mice when compared to wt littermates (Fig. 2B, D). Interestingly, it would therefore appear that the majority of *Abcc5*^*−/−*^ females can compensate to some degree for the loss of *Abcc5* (Fig. 2C) while male *Abcc5*^*−/−*^ mice are less able to do so as is reflected by the increased startle magnitude of male mice (Fig. 2D).

Furthermore, the response magnitude to the different levels of the prepulse cue alone (56, 58 and 65 dB) was measured in *Abcc5*^*−/−*^ and wt mice, for both females (Fig. 2E) and males (Fig. 2F). *Abcc5*^*−/−*^ females were significantly different to wt littermate controls for interaction (response magnitude x genotype, *P* < 0.0001) and response magnitude (*P* < 0.0001), but no significant genotype-dependent effect was seen (*P* = 0.89, Fig. 2E). Furthermore, the responses to all prepulse levels in *Abcc5*^*−/−*^ females were not significantly higher than that in wt controls (F_1,30_ = 0.0185, *P* = 0.89) (Fig. 2E). This would further support data observed in Fig. 2C, that the majority of *Abcc5*^*−/−*^ females can compensate to some degree for the loss of *Abcc5*. However, wt females displayed a gradient in their responses to increasing sound levels over 56 dB and the background response in wt females was 79.4 ± 12.7, followed by 64.3 ± 12.6, 153.7 ± 12.9, 161 ± 11.9 for 56, 58 and 65 dB, respectively, with a significant difference between 53–58 and 53–65 dB responses (P < 0.0001 for both). On the contrary, the response magnitude of *Abcc5*^*−/−*^ females to any tested prepulse sound level (56 dB, 58 dB or 65 dB) was not significantly different compared to background noise (53 dB) and averaged to 116 ± 12.

*Abcc5*^*−/−*^ males were significantly different for interaction (response magnitude x genotype, *P* < 0.0001), response magnitude (*P* < 0.004) and genotype (*P* = 0.0067) relative to wt littermate controls (Fig. 2F). Furthermore, the responses to all prepulse levels in *Abcc5*^*−/−*^ males were significantly higher than that in wt controls (F_1,26_ = 8.693, *P*= 0.0067) (Fig. 2F). Wt males show significantly higher responses to 58 and 65 dB sound levels of 129.6 ± 22 and 149.8 ± 15.6, respectively, compared to 53 dB background 81 ± 15.1 (*P* = 0.022 and *P* < 0.001), but not to the lowest prepulse level of 56 dB (67.9 ± 20). Similar to *Abcc5*^*−/−*^ females, *Abcc5*^*−/−*^ males showed no significant gradient response to 58 dB or 65 dB (175.9 ± 20.8 and 162.2 ± 12) when compared to the background (148.5 ± 16.4) levels, but did display an increased response magnitude to the lowest prepulse level of 56 dB (198.4 ± 22.9) vs background (*P* = 0.009) (Fig. 2F).

Notably, testing performed by the Mary Lyon Centre, UK as part of the International Mouse Phenotyping Consortium (recorded in the European Mouse Mutant Archive (EMMA), (https://www.mousephenotype.org/data/genes/ MGI:1351644) have reported no loss of auditory response in *Abcc5*^*−/−*^ mice, suggesting that changes in hearing are not a confounding factor in tests such as ASR and PPI.

In summary, *Abcc5*^*−/−*^ mice show a pronounced loss of PPI compared to wt littermate controls. However, the interpretation of the PPI results is complicated by the presence of ASR in combination with different variability in the data generated by wt vs *Abcc5*^*−/−*^ mice (i.e. different standard deviation in wt vs *Abcc5*^*−/−*^ mice). Overall, it would appear that wt animals can distinguish sounds louder than 56 dB and display a clear increase in response to 58 and 65 dB when compared to a 53 dB background pulse while, on the other hand, *Abcc5*^*−/−*^ mice showed an equal degree of reactivity throughout in response to 53–65 dB pulses.

### Abcc5^*−/−*^ mice show reduced cued fear expression in a fear conditioning paradigm

**Fig. 3:**
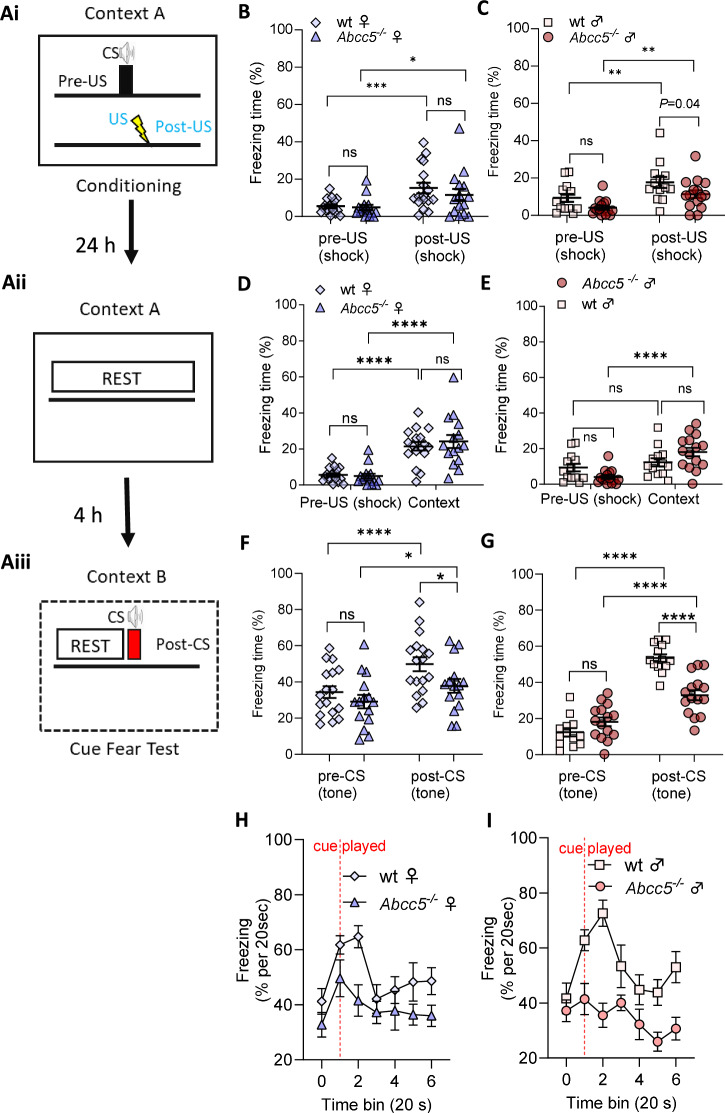
Fear conditioning in *Abcc5*^*−/−*^ mice show reduced cued fear expression. An outline of the fear conditioning protocol **Ai**–**Aiii**. The percentage of freezing during the conditioning trial session before (pre-US) and after (post-US) electric shock was applied to *Abcc5*^*−/−*^ (ko) and wt **B** female and **C** male mice. **D**, **E** The percentage of freezing during the contextual test 24 h later in the same testing chambers in **D** females and **E** males. **F**, **G** Percentage of freezing during cued fear conditioning test 4 h after contextual test shown before the tone was played (pre-CS) and after (post-CS) in **F** females and **G** males. Time of freezing is expressed as mean percentage ± SEM of total time for each test shown. **H**, **I** The percentage freezing normalised to freezing levels in the 20 s directly preceding the acoustic cue (red line – cue played) for **H** female and **I** male mice. Female: n_wt_ = 15, n_ko_ = 17 and male: n_wt_ = 15, n_ko_ = 13 mice. Each data point represents a measurement from an individual mouse **A**–**G** or average of n mice numbers per genotype ± SEM **H**, **I**. Results were compared by mixed-effect analysis combined with a Fisher’s LSD test for genotype comparison in the same treatment group, and a Šidák’s multiple comparison test when comparing genotypes across treatments; fixed effects: pre-and post-shock, genotype and pre-and post-shock x genotype; random effects: individual mice and residuals; ns (no significance), *P* < 0.05 *, *P* < 0.01 **, *P* < 0.001 ***, *P* < 0.0001 ****.

Learning and memory processes involve neural circuits such as the hippocampus, amygdala and prefrontal cortex which enable context-dependent behaviour [23]. To ascertain if memory and learning is affected by the loss of *Abcc5*, a fear conditioning (FC) test was conducted on 16-weeks old *Abcc5*^*−/−*^ mice and wt litter mate controls (Fig. 3). An overview of the fear conditioning protocol applied is shown in (Fig. 3Ai–Aiii, left panel). During the conditioning trial (Fig. 3Ai, B and C), mice exhibit an increased percentage of freezing in response to a mild electric shock, an aversive, unconditioned stimulus (US) that is applied directly after the conditioned stimulus (CS). The level of baseline, pre-US freezing for *Abcc5*^*−/−*^ and wt mice were similar (females: 4.9 ± 1.4% vs 5.5 ± 1.0%, Fig. 3B; males: 4.1 ± 1.1% vs 9.3 ± 2.1%, Fig. 3C). Freezing after foot-shock (post-US) increased in all mice to 11.6 ± 3.2% and 15.3 ± 2.9% in *Abcc5*^*−/−*^ and wt females and 11.4 ± 2.1% and 17.6 ± 2.9% in *Abcc5*^*−/−*^ and wt males (Fig. 3B, C). Therefore, the loss of *Abcc5* did not alter the fear response in mice pre- or post-US. The difference in freezing before (pre-US) and after (post-US) electric shock was significant in both females (F_1,30_ = 24.75, *P* < 0.0001, Fig. 3B) and males (F_1, 26_ = 27.03, *P* < 0.0001, Fig. 3C), but there was no significant difference between genotype (*P* = 0.446) in females, while genotype was significant for males (F_1,26_ = 5.02, *P* = 0.034). This suggests that mice of both genotypes responded similarly to the fear conditioning training, although male *Abcc5*^*−/−*^ animals presented a slightly reduced freezing response, as was similar to the phenotype found in PPI.

Contextual fear (Fig. 3Aii) was measured by placing mice in the same cages as for the conditioning trial and left to explore freely. Freezing was measured to assess the contextual fear triggered by the memory of an unpleasant experience from 24 h before in the same settings. No significant differences in freezing time between *Abcc5*^*−/−*^ mice and wt controls, for both female (Fig. 3D, Context) and male mice (Fig. 3E, Context) was detected (female *Abcc5*^*−/−*^ vs wt mice, 24.1%  ±  3.6 vs 21.4% ± 2.4; male *Abcc5*^*−/−*^ vs wt mice, 18.1% ± 2.4 vs 12.3% ± 2.3). Furthermore, the percentage freezing time was significantly increased for both genotypes and sexes when compared to the baseline (pre-US freezing levels during conditioning on Day 1; females, F_1,30_ = 72.79, *P* < 0.0001; males, F_1,26_ = 28.38, *P* < 0.0001). There was no genotype-related effect of contextual fear in females (F_1,30_ = 0.178, *P* = 0.67) or males (F_1,26_ = 0.014, *P* = 0.91) which would suggest that contextual fear processing is unaltered by the loss of *Abcc5*.

Conditioned association was assessed during an auditory cued fear test (Fig. 3Aiii). Mice were exposed to a new cage (Context B) which was visually different from the environment used in the fear conditioning trials (Context A). After a period of acclimatisation (pre-CS), a cue which was the same as CS was played and freezing time calculated post-CS.

Analysis of pre-CS freezing demonstrated that *Abcc5*^*−/−*^ mice show no difference in baseline freezing in either females (*P* = 0.2972, 29.1 ± 3.73% vs 34.36 ± 3.3%, for *Abcc5*^*−/−*^ vs wt. Fig. 3F) or males (*P* = 0.0968, 18.13 ± 2.44% vs 12.35 ± 2.26%, for *Abcc5*^*−/−*^ vs wt. Fig. 3G). In contrast, post-CS freezing was significantly reduced in *Abcc5*^*−/−*^ mice in both females (*P* = 0.0361, 37.81 ± 3.75 vs 49.81 ± 3.93 for *Abcc5*^*−/−*^ vs wt) and males (*P* < 0.0001, 32.91 ± 2.83 vs 53.52 ± 2.14 for *Abcc5*^*−/−*^ vs wt) (Fig. 3F, G). Although this genotype effect may imply that *Abcc5*^*−/−*^ mice have formed a weaker association between the US and CS, we noted that this may have been impacted by differences in the initial association between US and CS (Fig. 3B, C). We therefore analysed the data using a mixed effects model to compare the effect of genotype upon the difference between pre- and post-CS response. In both sexes the pre- to post-CS response had a significant effect (females: *P* < 0.0001, F_1,30_ = 29.54. Males: *P* < 0.0001, F_1,52_ = 127.8), indicating that the animals had formed an association between the US and CS. Male animals also showed a significant impact of genotype (F_1,52_ = 8.97, *P* = 0.004) and the interaction between genotype and the pre- to post-CS response (F_1,52_ = 28.64, *P* < 0.0001), confirming that *Abcc5*^*−/−*^ male animals have a reduced response to the CS. Conversely female animals showed no effect of the interaction between genotype and the pre- to post-CS response (F_1,30_ = 2.31, *P* = 0.14) and a non-significant trend in the effect of genotype alone (F_1,30_ = 2.3, *P* = 0.07), further suggesting that *Abcc5*^*−/−*^ female animals showed a significantly less pronounced phenotype than males.

To further substantiate this effect, we analysed freezing behaviour in 20 s bins, both pre- and post-CS, to identify at which time points in the response any genotype differences were evident (Fig. 3H, I). As expected, repeated measures analysis identified that both sexes showed a significant effect of time on freezing behaviour (females: *P* < 0.0001, F_5.1,153_ = 5.552. Males: *P* = 0.0006, F_4.5,118_ = 4.925). Males also showed a significant effect of genotype (*P* < 0.0001, F_1,26_ = 28.47) and the interaction between genotype and time (*P* = 0.0266, F_6,156_ = 2.46). Pairwise analysis indicated that *Abcc5*^*−/−*^ males showed significantly reduced freezing at time bin 0 (time of shock onset), 1, 4 and 5 (*P* < 0.05), suggesting a prolonged reduction in freezing response. Females showed a significant effect of genotype (*P* = 0.194, F_1,30_ = 6.099) but no significant interaction between genotype and time (*P* = 0.4518, F_6,180_ = 0.963), further highlighting the less pronounced phenotype in *Abcc5*^*−/−*^ females as compared to males.

In summary, the main difference between *Abcc5*^*−/−*^ mice and wt controls, for both females and males, is driven by the changes in the level of freezing post-cue. *Abcc5*^*−/−*^ mice freeze significantly less than their wt littermates and *Abcc5*^*−/−*^ mice response to the auditory cue was on average 12.0 ± 5.5% lower than wt for females, and 20.6 ± 3.6% for males post-CS, Fig. 3F, G).

### *Abcc5*^*−/−*^ mice show no changes in grip strength or open field behaviour

Muscle and motor function were tested in our mouse cohorts using a grip strength test, and locomotor and exploratory activity was tested using an open field test. In both phenotyping tests *Abcc5*^*−/−*^ mice showed no significant differences to wt littermates (Supplementary Fig. 2 & 3), suggesting that loss of Abcc5 does not impact these systems, with the exception of velocity (Supplementary Fig. 3I). Although female *Abcc5*^*−/−*^ mice covered the same distance in both the arena and centre (Supplementary Fig. 3A, G), they appeared to move faster when compared to wt littermates (Supplementary Fig. 3I).

### *Abcc5*^*−/−*^ mice show changes in the timing and fragmentation of sleep and activity rhythms

**Fig. 4:**
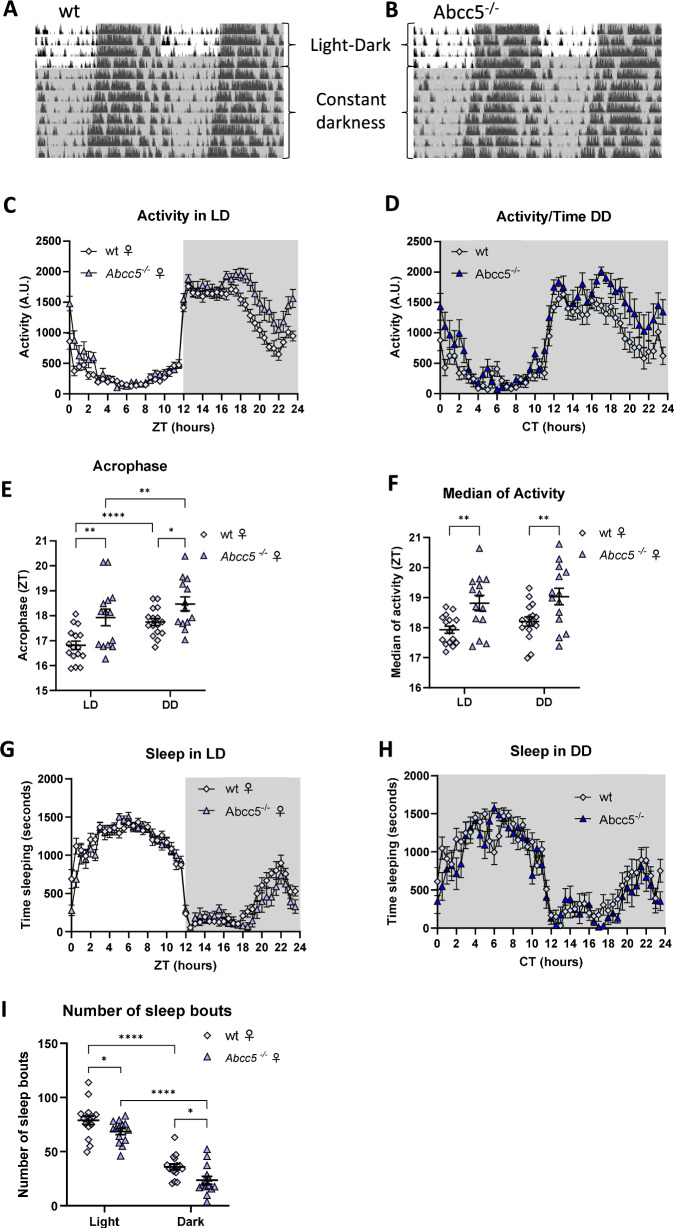
*Abcc5*^*−/−*^ mice show changes in the timing and fragmentation of sleep and activity rhythms. (**A**, **B** Representative double plotted actograms showing activity of wt **A** and *Abcc5*^*−/−*^ females **B** in light-dark cycles (LD) and constant darkness (DD). Vertical black lines represent animal activity and periods of darkness are indicated by grey shaded areas. **C**, **D** Mean activity levels over time in LD **C** and DD **D** conditions. **E** *Abcc5*^*−/−*^ animals show a delay in the acrophase (peak) of their activity rhythms. **F** *Abcc5*^*−/−*^ animals show a delay in the median of activity of their activity rhythms. **G**, **H** Mean time spent sleeping over time in LD **G** and DD **H** conditions. **I** *Abcc5*^*−/−*^ animals show a significant reduction in the number of sleep bouts in both the light and dark phases of LD. All above data generated from the same cohort of female mice: n_ko_ = 14, n_wt_ = 16. Data presented as mean ± SEM. Each data point is the cohort mean in **C**, **D**, **G**, **H**, and individual mice in **E**, **F**, **I**. Results were compared by mixed-effect analysis (REML) combined with a Fisher’s LSD test for genotype comparison; fixed effects: LD vs DD, genotype, LD vs DD x genotype; random effects: individual mice and residuals; *P* < 0.05 *, *P* < 0.01 **, *P* < 0.0001 ****.

Previous studies have demonstrated that *Abcc5*^*−/−*^ mice show increased activity in both the light and dark phases of the day [11]. Since such activity changes could impact upon circadian activity and sleep rhythms, we conducted further investigations using passive infrared monitoring of circadian activity and sleep in *Abcc5*^*−/−*^ female animals [19] (Fig. 4A, B). Activity analysis throughout a (12 h:12 h) light-dark (LD) cycle highlighted a significant effect of genotype (F_1,28_ = 15.70, *P* = 0.0005) and the interaction between genotype and time (F_47,1316_ = 2.83, *P* < 0.0001). Visual inspection of activity over time graphs indicated that *Abcc5*^*−/−*^ animals showed elevated activity levels at the end of the dark phase (Zeitgeber Time, ZT, 17.5 to 23.5) and in the early light phase (ZTs 0 to 2.5) (Fig. 4C), although, notably, no specific timebins were identified as significantly different following multiple test correction of pairwise comparisons. Analysis of the animals’ activity following their release into conditions of constant darkness (DD) highlighted similar results: genotype and the interaction between genotype and time were both significant factors affecting activity in DD (F_1,28_ = 22.81, *P* < 0.0001 and F_47,1316_ = 1.43, *P* = 0.0294 for genotype and genotype X time respectively). Although pairwise comparisons showed no significant differences at any specific timepoints, visual inspection suggested *Abcc5*^*−/−*^ animals showed elevated activity levels in the late subjective dark (Circadian Time, CT, 17–23.5) and early subjective light phases (CT 0–0.5 and 2) (Fig. 4D).

Consistent with these observations, circadian measures of the peak of activity rhythms (acrophase and the median of activity) [24] demonstrated that *Abcc5*^*−/−*^ animals, in both LD and DD conditions, showed a delay in the timing of their activity rhythm peak (*Abcc5*^*−/−*^ vs wt: LD acrophase: 17.93 ± 0.332 vs 16.82 ± 0.168, *P* = 0.0073; DD acrophase: 18.47 ± 0.277 vs 17.75 ± 0.141, *P* = 0.0308; LD median of activity: 18.82 ± 0.256 vs 17.93 ± 0.119, *P* = 0.0053; DD median of activity: 19.03 ± 0.275 vs 18.2 ± 0.154, *P* = 0.0159) (Fig. 4E, F). Other measures of circadian activity showed no significant differences, including measures of circadian period and the robustness of circadian rhythmicity (PN value, Intraday variability and Interdaily stability) (Supplementary Table S1).

Analysis of activity-defined sleep in LD conditions demonstrated that while sleep was not impacted by genotype alone (F_1,28_ = 4.095, *P* = 0.0526) there was a significant effect of the interaction between genotype and time (F_47,1316_ = 1.819, *P* = 0.0007) (Fig. 4G). Similar to the activity analysis above, visual inspection of graphs of sleep over time suggested that *Abcc5*^*−/−*^ mice showed reduced levels of sleep at the end of the dark phase (ZTs 20–21.5) and at the transition of lights turning on (ZTs 0–0.5), although these differences did not reach significance following multiple test corrections in pairwise comparisons. Activity-defined sleep analysis in DD highlighted a significant impact of genotype (F_1,28_ = 9.222, *P* = 0.0051), with *Abcc5*^*−/−*^ mice sleeping significantly less than wildtype controls (37009 ± 763 vs 32865 ± 1107 min sleeping over 24 h). However, in DD conditions there was no significant effect of the interaction between sleep and time (F_47,1316_ = 1.192, *P* = 0.1773) (Fig. 4H). Sleep fragmentation was investigated through analysis of sleep bouts. A sleep bout is defined as an uninterrupted period of sleep (e.g., from the time at which sleep initiates to the time of waking). Analysis of the average length of sleep bouts demonstrated no significant difference in *Abcc5*^*−/−*^ mice compared to controls in either the light or dark phases (light phase: *P* = 0.6962; dark phase: *P* = 0.3787). However, analysis of the total number of sleep bouts demonstrated that *Abcc5*^*−/−*^ animals showed a significantly fewer sleep bouts in both the light and dark phases (*Abcc5*^*−/−*^ vs wt: light phase: 68.48 ± 2.79 vs 78.91 ± 3.95, *P* = 0.0451; dark phase: 23.43 ± 3.586 vs 35.78 ± 2.665, *P* = 0.0106) (Fig. 4I). The sleep and circadian data we present here are limited to female animals, due to animal welfare concerns regarding prolonged single housing of male mice.

### *Abcc5*^*−/−*^ mice exhibit altered synaptic transmission

Previous work showed that brain tissue from *Abcc5*^*−/−*^ mice exhibited a 2.4-fold increase in NAAG levels [4], suggesting that Abcc5 regulates NAAG availability in vivo. In order to directly ascertain if the loss of Abcc5 in rodent brain tissue affects synaptic transmission, electrophysiological recordings were performed on brain slices from *Abcc5*^*−/−*^ mice and compared to wt.

**Fig. 5:**
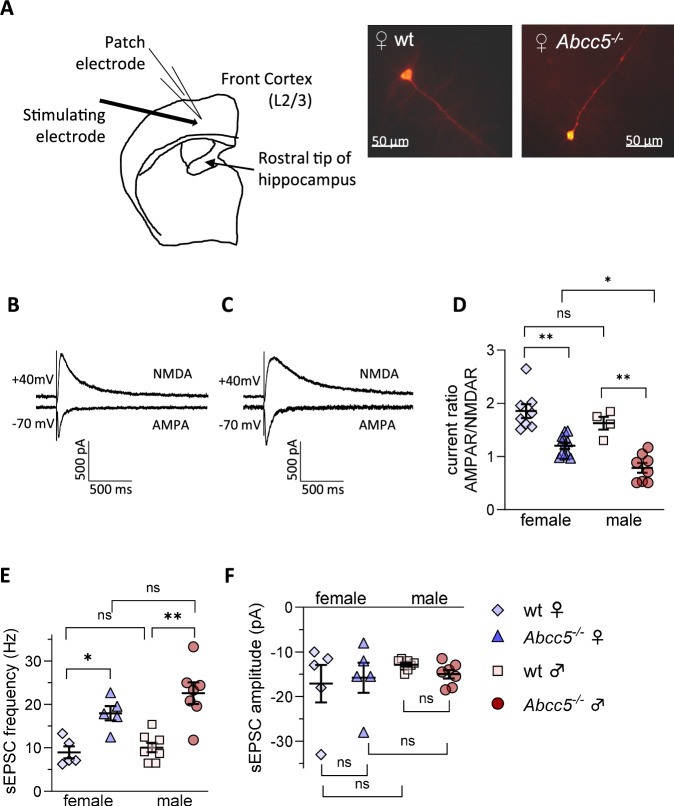
Electrophysiology analysis. **A** Diagram of the recording configuration and placement of the stimulation electrode to activate cortical afferents (left). Example fluorescence images of pyramidal cells used for patch recordings (right). **B**, **C** Representative traces of evoked cortical EPSCs, from acute brain slice of wild-type **B** and *Abcc5*^*−/−*^ **C** littermate male mice. **D** Ratio of AMPAR:NMDAR peak currents in frontal cortex pyramidal neurons stimulated with 100–200 μA for 100 μs, females: n_ko_ = 11, n_wt_ = 8 and males: n_ko_ = 8, n_wt_ = 4 Spontaneous EPSC **E** frequency, females: n_ko_ = 5, n_wt_ = 5 and males: n_ko_ = 7, n_wt_ = 8 **F** amplitude, n_ko_ = 5, n_wt_ = 5 and males: n_ko_ = 7, n_wt_ = 8. Brain slices were from 3 mice per sex and phenotype, aged 12–16wks. All data analysed by Welch and Brown-Forsythe one-way ANOVA in combination with a Dunnet multiple comparison post-hoc test. Data shown as mean ± SEM. ns, no significance; *P* ≤ 0.05 *, *P* ≤ 0.01 **.

Patch clamp recording from pyramidal cells in the 2/3 layer of the frontal cortex (Fig. 5A–C), showed altered synaptic AMPA/NMDA receptor current ratios (AMPAR, α-amino-3-hydroxy-5-methyl-4-isoxazolepropionic acid receptor; NMDAR, N-methyl-D-aspartate receptor) in both female and male *Abcc5*^*−/−*^ mice when compared to wt littermates (female: 1.20 ± 0.06 vs 1.86 ± 0.13, *P* = 0.006; male: 0.79 ± 0.09 vs 1.63 ± 0.12, *P* = 0.004; Fig. 5D). There was no difference between sexes in wt mice, but female *Abcc5*^*−/−*^ mice had a significantly higher ratio of AMPA/NMDA receptor current than *Abcc5*^*−/−*^ male mice (*P* = 0.013). Furthermore, an increased frequency of spontaneous excitatory postsynaptic currents (sEPSC) was observed in *Abcc5*^*−/−*^ mice of both sexes when compared to wt littermates (female: 17.98 ± 1.67 vs 8.97 ± 1.37, *P* = 0.016 and male: 22.6 ± 2.45 vs 10.06 ± 1.08, *P* = 0.008) (Fig. 5E). No significant differences were observed between sexes for both *Abcc5*^*−/−*^ or wt mice. No changes in current amplitude were observed between genotype or sex (−15.8 ± 3.35 vs −17.1 ± 4.2 for females and −15.0 ± 0.96 vs −12.88 ± 0.41 for males, Fig. 5F). Generally, changes in the frequency of sEPSCs is indicative of increased presynaptic release mechanisms, while changes in current amplitude of sEPSCs would suggest altered postsynaptic response mechanisms. As frequency, but not current amplitude, of sEPSCs are changed in *Abcc5*^*−/−*^ mice, electrophysiological data would suggest that loss of Abcc5 has a presynaptic impact.

## Discussion

Abcc5 is an orphan ATP-binding cassette transporter that is reported to export structurally unrelated compounds including various anti-cancer drugs, glutamate conjugates, such as N-acetyl-aspartyl-glutamate (NAAG), N-acetyl-aspartyl-glutamyl-glutamate (NAAG_2_), and beta-citryl-glutamate (BCG), as well as cyclic nucleotides and haem [4, 25–27].

The literature on rodent Abcc5 and human ABCC5 dovetails into studies which investigates the loss of function of Abcc5 in knock-out mouse models or the regulation at gene expression level in human cancers. Gain-of-function genetic studies all point to an important role for human ABCC5 in chemotherapy resistance, while knock-out models suggest a different physiological role for Abcc5 in adipose tissue, the brain, testis, gut formation, haem metabolism and the eye [4, 11, 26–29].

Here we report the neurobehavioral profiling of *Abcc5*^*−/−*^ mice. The loss of Abcc5 affected both male and female mice behaviour and both sexes had a profound loss of prepulse inhibition (PPI, Fig. 1), with male mice having a more pronounced phenotype. However, the interpretation of these results is complicated by the presence of prepulse facilitation (PPF), and the fact that *Abcc5*^*−/−*^ mice cannot discern between different sound levels (Fig. 2), which would indicate that multiple systems are affected by the loss of Abcc5. Female mice also appear to be less affected by the loss of *Abcc5*^*−/−*^ and the majority of *Abcc5*^*−/−*^ female mice appear to be able to compensate for the loss of *Abcc5*^*−/−*^ to some degree (Fig. 2). *Abcc5*^*−/−*^ mice also demonstrate a substantial decrease in fear conditioning responses (Fig. 3) which further suggests that the circuits needed for the tight temporal control essential to attention processes are affected in *Abcc5*^*−/−*^ mice. Analysis of circadian activity rhythms suggested that *Abcc5*^*−/−*^ mice show a delay in the peak of their activity cycle (Fig. 4C, D). In addition to this, analysis of sleep highlighted that *Abcc5*^*−/−*^ mice sleep less than wt littermates (Fig. 4E, F). Notably, despite this reduction in the total amount of sleep, *Abcc5*^*−/−*^ mice showed significantly lower numbers of sleep bouts, suggesting a less fragmented sleep pattern (Fig. 4I). It is possible that this reduction in the number of sleep bouts was a compensatory mechanism against the loss of total sleep in these animals. However, further studies highlighting sleep effects in *Abcc5*^*−/−*^ male mice, which appears to have a more pronounced phenotype, would be of great interest. Patch-clamp experiments on both sexes showed that NMDAR current amplitude is affected by the loss of *Abcc5*^*−/−*^ and that spontaneous excitatory postsynaptic current (sEPSC) frequency, but not amplitude, is increased in *Abcc5*^*−/−*^ mice. This would suggest that presynaptic processes are affected by the loss of Abcc5 activity, but do not definitively rule out the possibility that both pre- and postsynaptic processes are affected by the loss of Abcc5 (Fig. 5).

Our behavioural characterisation of *Abcc5*^*−/−*^ mice highlighted the potential diversity of neuronal networks which are regulated by Abcc5 function. Similar phenotypes have also been observed in mouse models of conditions such as schizophrenia, autism spectrum disorder and Dravet syndrome [30–32]. Similarly to *Abcc5*, the genes targeted in such models are not limited to specific nuclei or regions of the brain, highlighting that such phenotypes may be the result of multi network changes across the brain. Furthermore, although ABC transporters have thus far not been implicated in the regulation of glutamatergic signalling, we do note that the phenotypes we observe here have all been previously demonstrated to be modulated by NMDAR function, either through pharmacological interventions, or genetic studies [33–36].

Previous studies indicated that brain tissue from *Abcc5*^*−/−*^ mice exhibit a 2.4-fold increase in NAAG, suggesting that Abcc5 likely transports NAAG in vivo or alternatively, affect the synthesis or breakdown of NAAG and NAAG_2_ via GPCR signalling cascades similar to Abcc4 [4]. These findings are supported by the data reported here of altered NMDA receptor currents in our *Abcc5*^*−/−*^ mice.

NAAG is a neuropeptide that modulates glutamatergic signalling by activating an inhibitory presynaptic metabotropic receptor mGluR3, which decreases neurotransmitter release and mediates negative feedback to protect against excessive glutamate release during periods of intense neuronal activity [37]. NAAG has also been shown to modulate postsynaptic excitatory NMDA receptor (NMDAR) currents, though contradicting studies have shown that NAAG may activate or inhibit NMDAR depending on ligand concentration [8, 38–40]. Although a possible role for NAAG in schizophrenia pathology has been proposed, the exact mechanism is not well understood and remains controversial [8, 41, 42]. It was proposed that NMDA receptor antagonists are associated with increased glutamate release due to the differential sensitivity of NMDA receptors on the rapid-firing, recurrent, parvalbumin-positive GABAergic interneurons, resulting in pyramidal neuron disinhibition [43].

It is important to note that neither NAAG or NAAG_2_, nor the enzymes involved in their synthesis or breakdown, have been linked to schizophrenia by recent GWAS studies [44]. However, *GRM3* (the gene encoding the mGluR3 receptor) has been associated with schizophrenia and with psychosis [45–47] and altered expression have been reported in the brains of schizophrenic patients [47]. Additionally, mGluR3 has been demonstrated to regulate both cognition and sleep [48, 49]. It is clear from the data reported here that the loss of rodent Abcc5 has a neurological impact which would correlate with reported increased levels of NAAG observed in the brain of *Abcc5*^*−/−*^ mice and altered NMDA receptor activity reported here. In support of this finding, several GWAS and gene network expression studies have identified *Abcc5* gene expression to be important in the normal function of the brain [14, 15, 50, 51]. It is well documented that the loss of an ABC transporter can be compensated for by the upregulation of a related transporter, and knock-out studies have demonstrated that, in mice, Abcc5 and Abcc12 can compensate for the loss of the other [25]. It is therefore feasible that the loss of ABCC5 in humans can be compensated for by the closely related family members ABCC11 and ABCC12.

An important limitation of the current study is that we do not know where in the brain, and in which neurons, NAAG accumulates in *Abcc5*^*−/−*^ mice. The temporal and spatial accumulation of glutamate dipeptides in *Abcc5*^*−/−*^ mice will determine the neurological impact of the loss of Abcc5. Furthermore, the underlying molecular mechanism of how Abcc5 activity regulates NAAG availability remains unknown and needs further investigation.

A recent study reported the high-resolution structure of human ABCC5, with a central density in agreement with a peptide positioned in the substrate binding pocket [52]. As the model cell line used to overexpress ABCC5 for these structural studies, HEK cells, also express the enzymes which drives the synthesis of NAAG and NAAG_2_, the possibility that ABCC5 was found with its physiological ligand bound cannot be ruled out (https://www.proteinatlas.org/ENSG00000166532-RIMKLB/cell+line). Importantly, the study reports the synthesis of peptide inhibitors with the potential to treat ABCC5-driven drug resistance in cancer patients.

In conclusion, the physiological substrates and function of Abcc5 in mammals remain elusive, and a mechanistic understanding of physiological role of this transporter is still lacking. However, as the loss of Abcc5 in rodents leads to a clear neurobiological phenotype, it would be prudent to not use ABCC5 inhibitors to treat multidrug resistant tumours in humans before the physiological substrate and function of ABCC5 is thoroughly characterised, as our data suggest that the inhibition of ABCC5 in the brain may lead to unexpected neurological side-effects.

## Supplementary information

Supplemental Material

## Data Availability

Any raw data would be made available upon request from the corresponding author.

## Reporting and reproducibility

The authors confirm that all methods were performed in accordance with the relevant guidelines and regulations set by the University of Oxford’s best practise guidance as well as the journal’s Guide to Authors, and is further stipulated in the Reporting Summary.
