# Supplementary material for: The murine ATP-binding cassette transporter C5 (Abcc5/MRP5/cMOAT) plays a role in memory consolidation, circadian rhythm regulation and glutamatergic signalling
Source: Transl Psychiatry. 2025 Jul 1;15:218. doi: 10.1038/s41398-025-03438-9 (PMC12214961; doi:10.1038/s41398-025-03438-9)
Supplement: Supplementary file 1 — Supplemental Material [file 41398_2025_3438_MOESM1_ESM.pdf]

Supplementary Figures

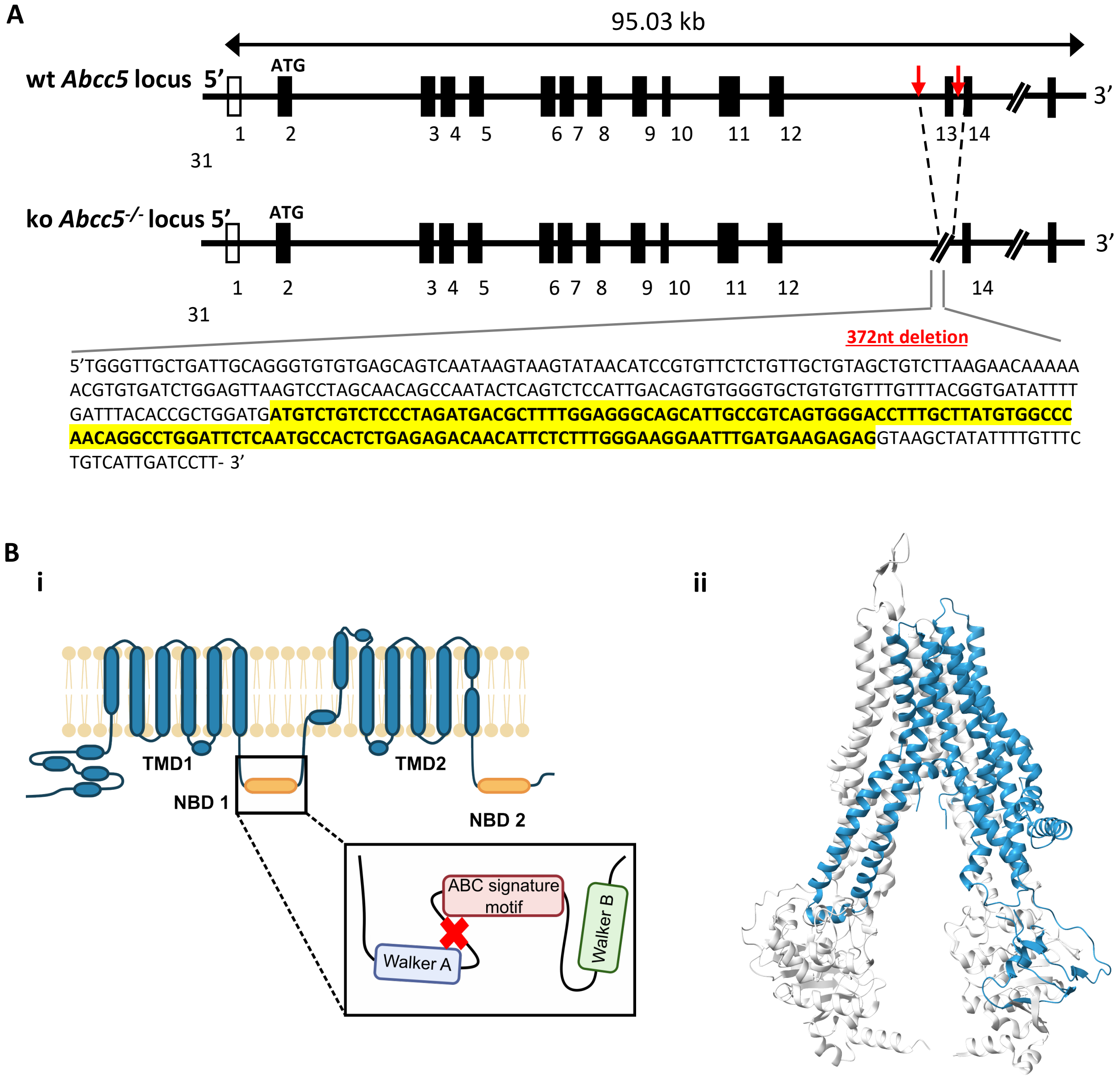

SFigure 1. CRISPR deletion of ATP-binding cassette transporter C5 (Abcc5) ko mice (A) Genomic overview of *Abcc5*$^{-/-}$ CRISPR/Cas9 knock-out design showing the 372 nucleotides deletion resulting in full ablation of exon 13 as well as a frame shift to produce a premature stop codon of the remaining coding sequence. Exon 13 is coding amino acids 612-646aa corresponding to the nucleotide binding domain 1 (NBD1) region of the protein. **(B) (i)** Topological overview of Abcc5 protein showing two transmembrane domains (TMD1 and TMD2) with their corresponding nucleotide binding domains (NBD1 and NBD2). CRISPR/Cas9 deletion introduced on a C57BL/6NTac background is shown by red cross. **(ii)** Structural representation of human ABCC5[53] (PDB: 8WI0) with the deletion that would result from the 372 nucleotide deletion and premature stop codon of the remaining coding sequence shown in grey. Figure prepared using UCSF ChimeraX.

Chimera Reference: Goddard, T. D. et al. UCSF ChimeraX: meeting modern challenges in visualization and analysis. *Protein Sci.* **27**, 14–25 (2018)

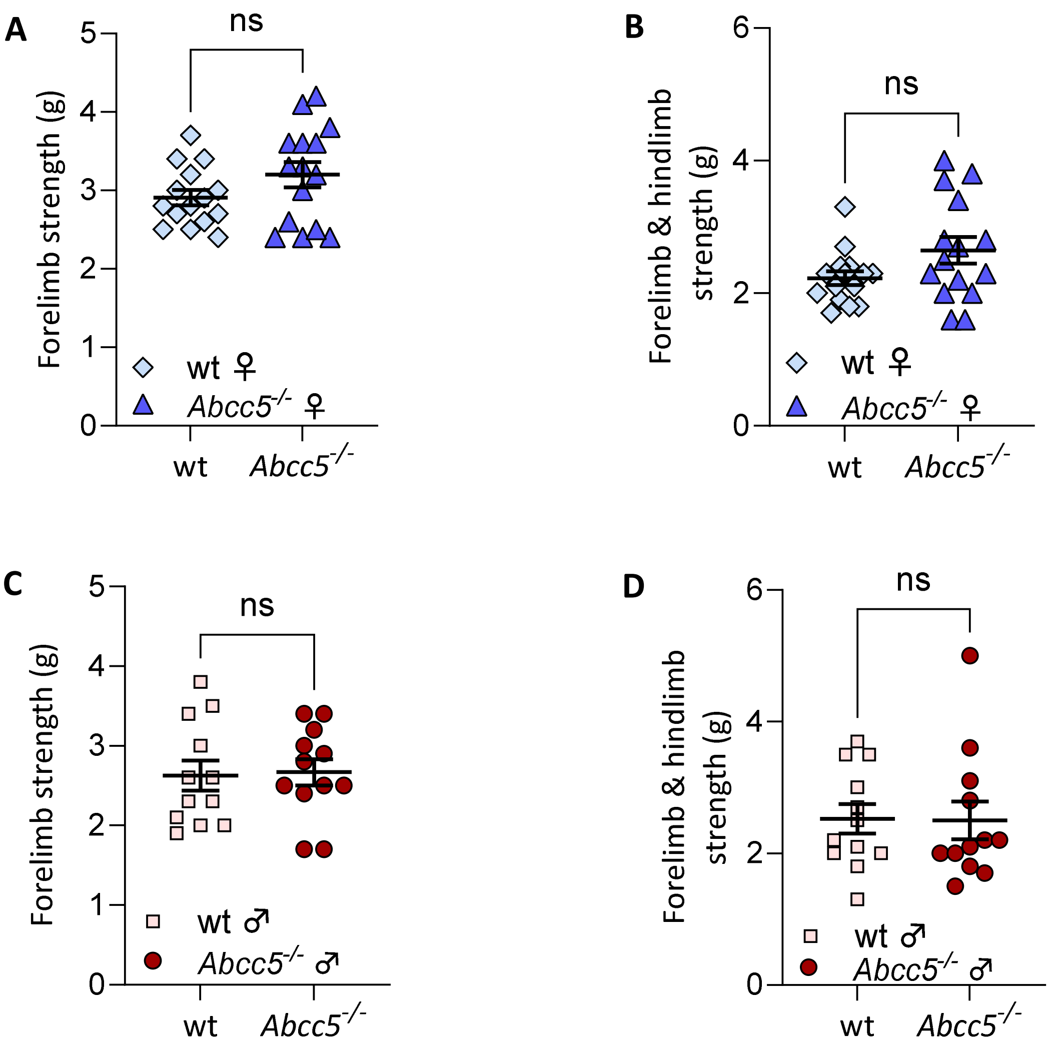

SFigure 2. Grip strength of *Abcc5*$^{-/-}$ mice is similar to wt. (**A&C**) Forelimb and **(B&D)** combined forelimb and hindlimb grip strength for *Abcc5*$^{-/-}$ and wt mice. **(A&B)** females (n_{wt}=15, n_{ko}=15) and **(C&D)** males **(**n_{wt}=12, n_{ko}=12) expressed per g of body weight. Each data point represent an average of 3 measurements from a single mouse±SEM. Data were analysed using an unpaired student's t-test; ns (no significance). Grip strength was assessed using a Grip Strength Meter (BioSeb, Chaville, France). Readings were taken from both forelimb only and combined forelimb and hindlimbs. Grip strength readings were taken in triplicate, then averaged and normalized to body weight. Animals were tested at 14 weeks of age.

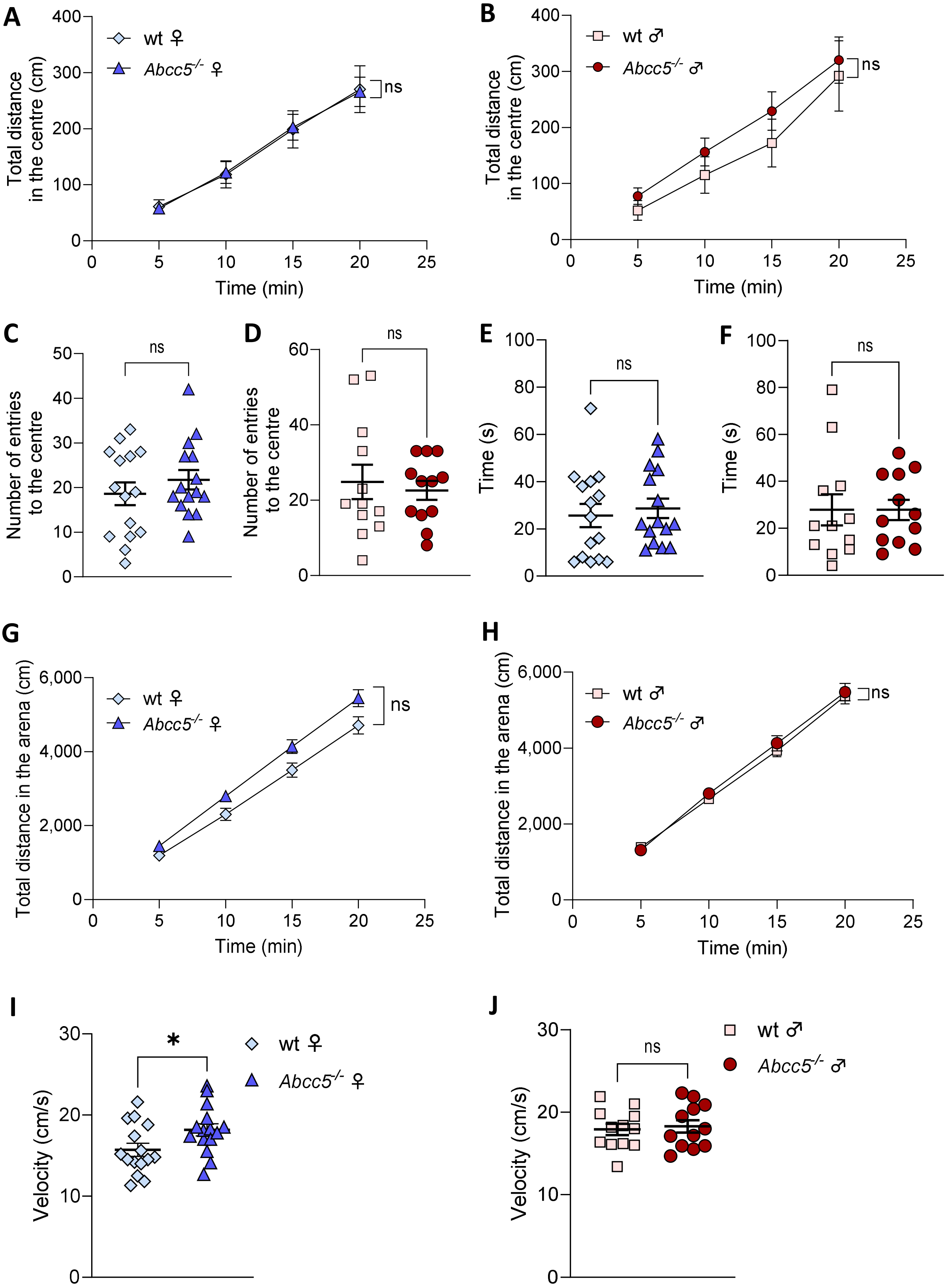

A
Total distance in the centre (cm)
Time (min)
wt ♀
Abcc5-/- ♀
ns
B
Total distance in the centre (cm)
Time (min)
wt ♂
Abcc5-/- ♂
ns
C
Number of entries to the centre
ns
D
Number of entries to the centre
ns
E
Time (s)
ns
F
Time (s)
ns
G
Total distance in the arena (cm)
Time (min)
wt ♀
Abcc5-/- ♀
ns
H
Total distance in the arena (cm)
Time (min)
wt ♂
Abcc5-/- ♂
ns
I
Velocity (cm/s)
*
wt ♀
Abcc5-/- ♀
J
Velocity (cm/s)
ns
wt ♂
Abcc5-/- ♂

SFigure 3. *Abcc5$^{-/-}$* mice show no change in anxiety-like behaviour or general locomotor activity in an Open Field test. (A&B) Distance (cm) travelled in the centre of the Open field arena plotted as cumulative function of the time in 5min time-bins for *Abcc5$^{-/-}$* mice (ko) and wt controls, females (n_{wt}=15, n_{ko}=15) and males (n_{wt}=12, n_{ko}=12) **(C&D)** The number of total entries into the centre of the arena in **(C)** females and **(D)** males. **(E&F)** Total time spent in the centre of the arena within 20min duration of the test for **(E)** females and **(F)** males. **(G&H)** Total distance (cm) travelled in the whole arena plotted as cumulative function of the time in 5min time-bins in **(G)** females and **(H)** males of both genotypes. **(I&J)** Velocity of movement (cm/s) in the whole arena of **(I)** females and **(J)** males. Each point is a mean from n number of mice±SEM for A and B, G and H. Each data point represents a single mouse±SEM for C-F and J-I . Data were analysed using an unpaired Student's t-test; ns (no significance), $P \leq 0.05$ *.

Open-field activity was used to assess locomotion and exploration in a novel environment. Mice were placed into one corner of a walled arena (grey polyvinyl chloride; 45 × 45 cm; light levels of 150-200 lux) and allowed to freely explore for 20 min. The arena is divided in two zones of exploration: the periphery and the centre (12 cm × 12 cm). Animal movements and position within the arena were tracked using EthoVision XT analysis software (Noldus) and parameters such as distance moved and duration in different zones over the entire 20-min period was recorded. Animals were tested at 10 weeks of age.

Supplementary Table 1. Circadian analysis.

	Amplitude (PN)		Intradaily variability		Interdaily stability		Period (hours)
	LD	DD	LD	DD	LD	DD	
**wt**	1168 ±82	2140 ±144	0.678 ±0.004	0.855 ±0.004	0.698 ±0.023	0.667 ±0.018	23.77 ±0.027
**Abcc5$^{-/-}$**	1372 ±113	2037 ±238	0.615 ±0.005	0.911 ±0.005	0.685 ±0.033	0.618 ±0.022	23.75 ±0.037
**P**	0.149	0.705	0.319	0.405	0.761	0.091	0.364

Supplementary Table 1. Analysis of the robustness of the circadian rhythms of *Abcc5$^{-/-}$* mice (as measured by circadian amplitude, intradaily variability and interdaily stability) demonstrated no significant differences from wildtype animals in either LD cycles or conditions of constant darkness. Similarly, there was no different in circadian period in *Abcc5$^{-/-}$* mice when compared to wildtype controls.
